# Changes in depression and suicidal ideation under severe lockdown restrictions during the first wave of the COVID-19 pandemic in Spain: a longitudinal study in the general population - RETRACTION

**DOI:** 10.1017/S2045796023000082

**Published:** 2023-03-13

**Authors:** J. L. Ayuso-Mateos, D. Morillo, J. M. Haro, B. Olaya, E. Lara, M. Miret

**Affiliations:** 1 Department of Psychiatry, Hospital Universitario de La Princesa, Instituto de Investigación Sanitaria Princesa (IIS- Princesa), Madrid, Spain; 2 Instituto de Salud Carlos III, Centro de Investigación Biomédica en Red de Salud Mental, CIBERSAM, Madrid, Spain; 3 Department of Psychiatry, Universidad Autónoma de Madrid, Madrid, Spain; 4 Parc Sanitari Sant Joan de Déu, Universitat de Barcelona, Sant Boi de Llobregat, Barcelona, Spain

The authors would like to retract the entire article above based on a genuine error noticed after publication. More precisely, as part of further analysis with the data set, they discovered the depression variables were inadvertently mislabeled (e.g., lifetime depression was labeled as 12-months depression). Consequently, the results and conclusions of the article are significantly affected.

On the one hand, the prevalence of depression increased significantly. On the other hand, the analyses that explored the factors associated with the incidence of depression and suicidal ideation have also been affected. In the final regression model for depression, the coefficient for COVID-19 concern was found to be significant, along with the post-measures of loneliness and resilience. In addition, the final regression model for suicidal ideation showed significant associations for the Post-measures variables of social support and disability.

The authors apologize for the inconveniences that this incident may have caused to the readers. A new corrected version of the research will be resubmitted and resupplied later.
